# Initial Experience of Transforaminal Lumbar Endoscopic Discectomy in a Single Orthopedics Unit

**DOI:** 10.7759/cureus.91589

**Published:** 2025-09-04

**Authors:** Faisal Hamad, Muhammad Mansha, Malik S Ahmed, Housamaddeen Alfarhan, Abdel Rehim El Tayeh

**Affiliations:** 1 Orthopaedics, Hamad Medical Corporation, Doha, QAT; 2 Orthopaedics Surgery, Hamad Medical Corporation, Doha, QAT

**Keywords:** endoscopic lumbar discectomy, lumbar disc surgery, lumbar spine surgery, minimally access surgery, spine endoscopy

## Abstract

Objectives

The objective of this study is to report the initial surgical experience, challenges faced, and the preliminary outcomes of our cases.

Materials and methods

A retrospective analysis of the first 10 patients operated with TELD in our unit was performed. Ethical approval was obtained from the Hospital Review Committee of Hamad Medical Corporation. Preparation time and surgical time were used to determine the learning curve of the surgeons. The Oswestry Disability Index (ODI) scores, which are routinely recorded preoperatively and then at two weeks and three months postoperatively, were used to assess the outcomes in this study. All the data was extracted from patients' records. The procedures were performed by two orthopedic spine surgeons. A total of 12 cases were booked for the TELD from April 2023 to February 2024. Two cases were converted to microscopic discectomy intraoperatively due to difficult access, and they were excluded from the study.

Results

Ten cases underwent TELD at the (L3/L4 - 1 case, L4/L5 - 8 cases and L5/ S1- 1 case) levels. All patients were male, and the mean age was 37.9 years (range: 24 to 48 years). Preparation time remained almost stable, while a downward trend was noticed in surgical time after the fourth case. The first and eighth operated cases did not show significant improvement in the ODI scores. The remaining patients had significant improvement in ODI scores, especially at three months postoperatively.

Conclusion

By virtue of our preliminary experience of the TELD, we realized that it is a useful procedure in properly selected patients with all the benefits of minimally invasive surgery. However, we found a relatively late resolution of preoperative symptoms as indicated by the ODI scores.

## Introduction

Disc herniation (Dh) is one of the common causes of back pain and lower limb pain [1]. Due to failure of conservative treatment [2] and in the presence of a neurological deficit, surgery is usually indicated.

In 1934, Mixter and Barr started to operate Dh by doing laminectomy with open discectomy [3]. Later on, this procedure was performed with the help of a microscope and was named microscopic discectomy (Md) [4]. Kambin was the first surgeon to explain the endoscopic procedure for a herniated disc [5]. Endoscopic discectomy was described for the first time in 1997 [6].

Although Md is still considered the gold standard procedure for the surgical treatment of lumbar Dh [1], transforaminal endoscopic lumbar discectomy (TELD) has recently become more popular among spine surgeons around the globe [6].

Reduced trauma to soft tissue, resulting in decreased blood loss intraoperatively, lesser pain postoperatively, reduced stay in hospital, and speedy recovery with sooner return to routine activities [7,8] are some of its benefits. Amongst the potential drawbacks are inadequate discectomy, elevated recurrence rate, a prolonged surgeon’s learning curve [9], and being much subject to radiation intraoperatively [10].

The fundamental indications for TELD are (i) soft Dh as manifested on MRI and CT scans, (ii) definitive lumbar radiculopathy compatible with the radiological findings, and (iii) failure of nonoperative treatment for at least six weeks. The contraindications for TELD are (i) severe central stenosis, (ii) segmental instability, (iii) painless weakness, (iv) cauda equina syndrome, and (v) tumor or infection [11].

At our institution, HMGH-HMC we have recently started TELD to treat patients with Dh. In this retrospective case series, we share our initial experience including technical challenges, learning curve, and early patient outcomes.

## Materials and methods

A retrospective analysis of the first 10 patients operated with TELD in our unit was performed. Those patients who were booked for Md were not included in this study. Ethical approval was obtained from the Hospital Review Committee of Hamad Medical Corporation. The Oswestry Disability Index (ODI) scores, which are routinely recorded preoperatively and then at two weeks and three months postoperatively, were used to assess the patient's satisfaction and outcomes in this study. All the data was extracted from patients' medical records. The procedure was performed by two orthopedic spine surgeons. A total of 12 cases were booked for the TELD from April 2023 to February 2024. However, two cases were converted to Md intraoperatively due to difficult access, and they were excluded from the study. Preparation time and surgical time were used to determine the learning curve of the surgeons. Preparation time was defined as the time period from anesthetizing the patient to the start of incision, which includes patient positioning, draping, and setting for surgery, and surgical time was defined as the time period from skin incision to wound closure.

ODI scores

Introduced in 1980, ODI [12] is a questionnaire answered by patients. The 10 ODI questions for assessment include the magnitude of pain, difficulty in personal daily care, lifting, routine work, various positions including sitting, standing, and sleeping, sexual and social life, and ease at travel [13].

Each single question is scored from 0 to 5, with a maximum of 50. This score is then multiplied by 2 and then classified as: 0-20, minimal disability; 21-40, moderate disability; 41-60, severe disability; 61-80, severe back pain; 81-100, bed-bound. A change in the patient’s score of 10% or more is considered significant [13].

Surgical technique

**Figure 1:**
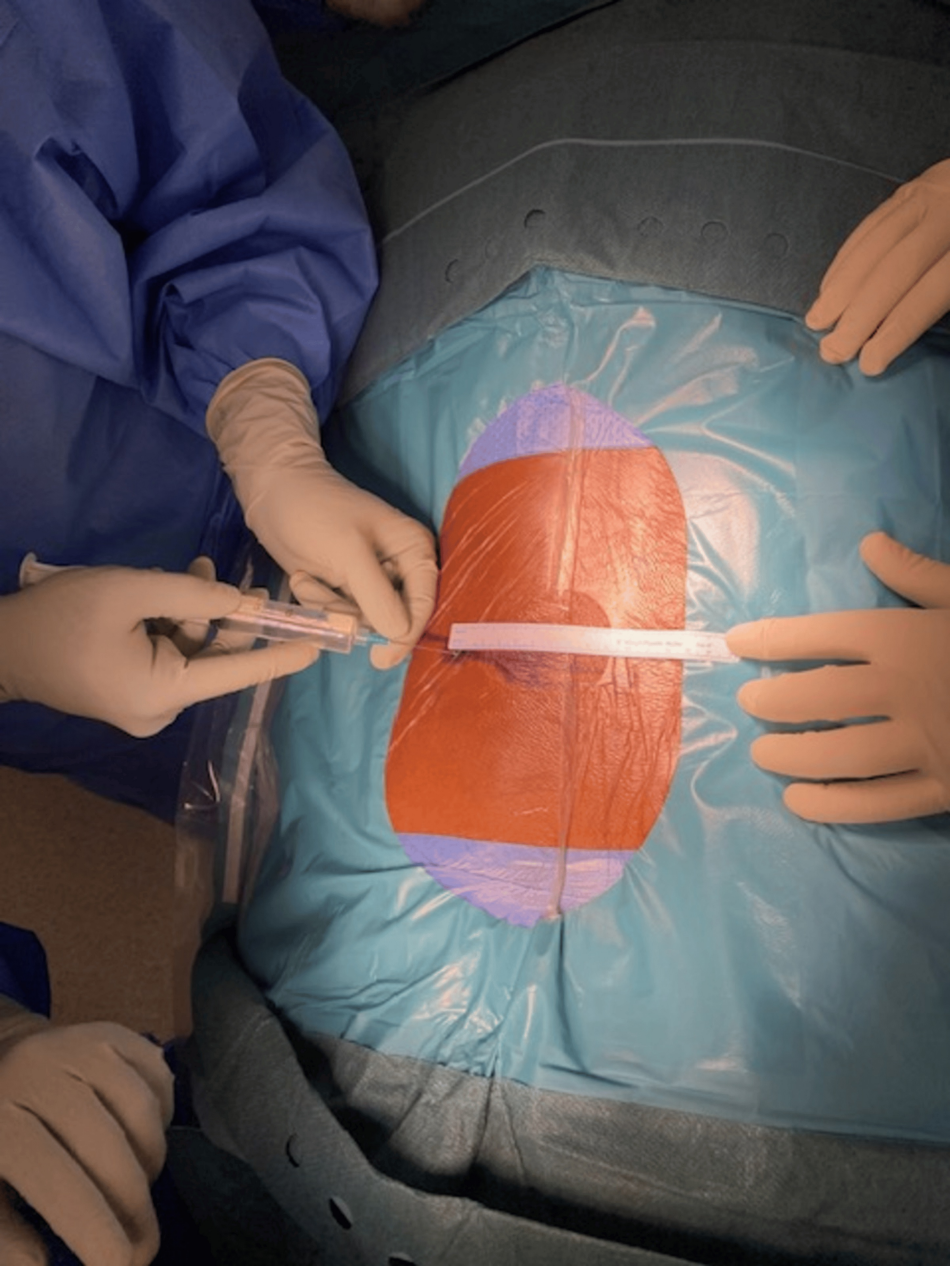
Marking for the entry point around 10 to 12cm lateral to the midline

**Figure 2:**
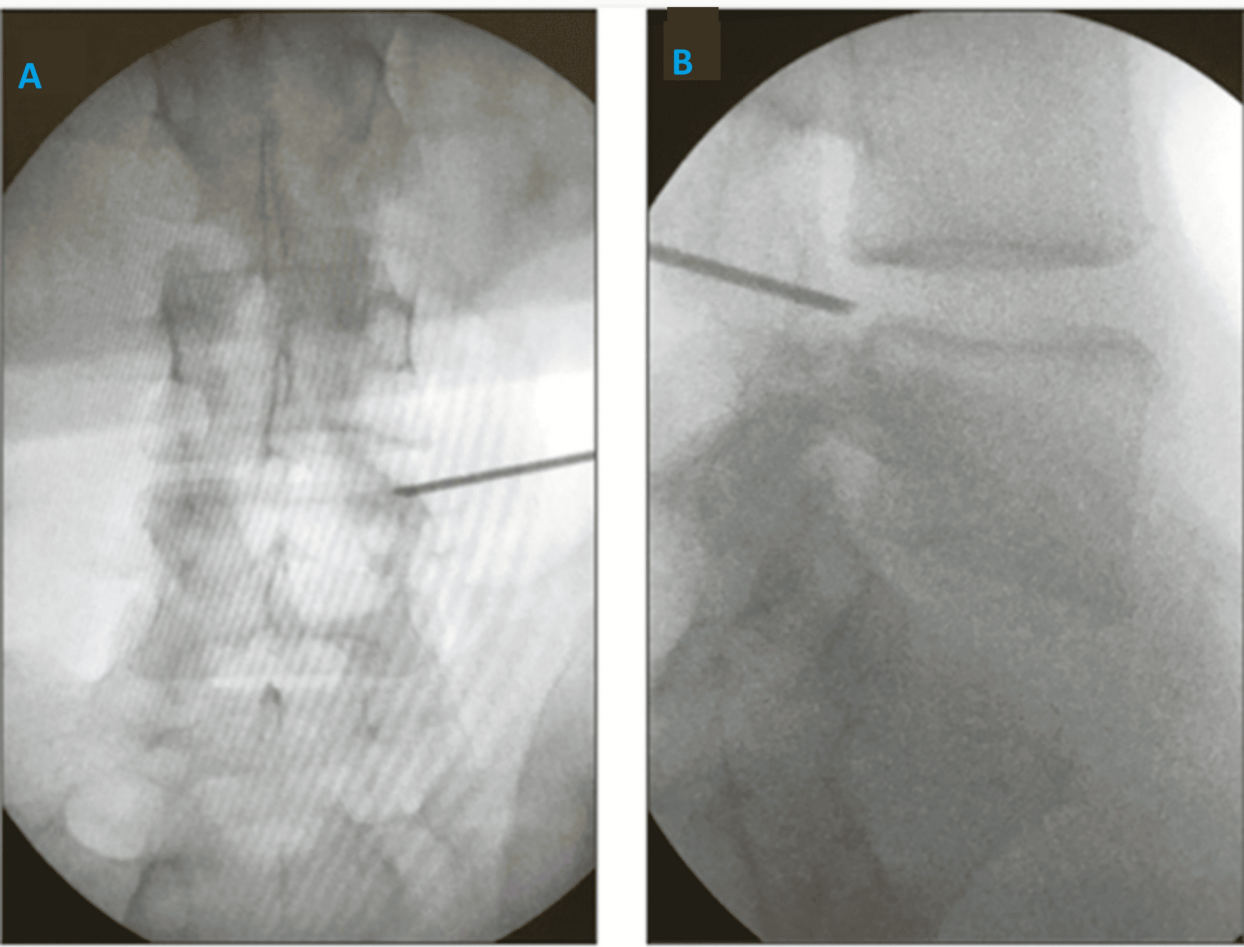
Needle tip between medial and lateral pedicular lines in AP view (A) and at the posterior vertebral line in the lateral view (B) AP: Anteroposterior

**Figure 3:**
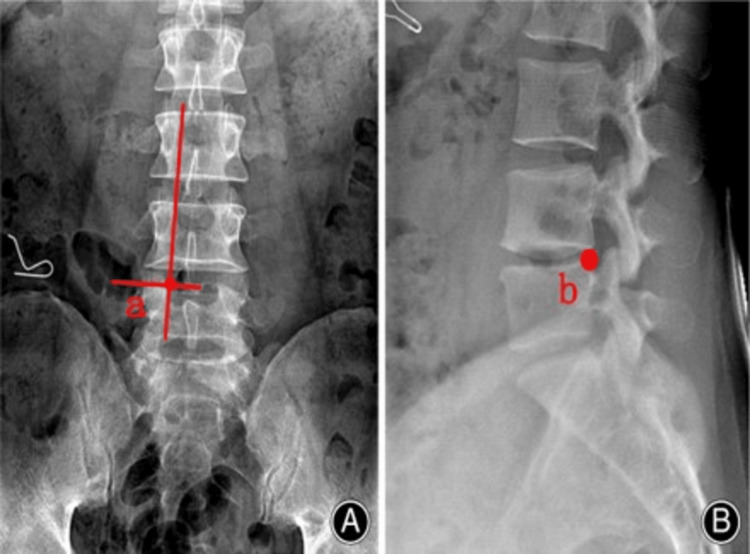
Target point is the medial pedicular line (A) upper corner of caudal vertebra (B)

**Figure 4:**
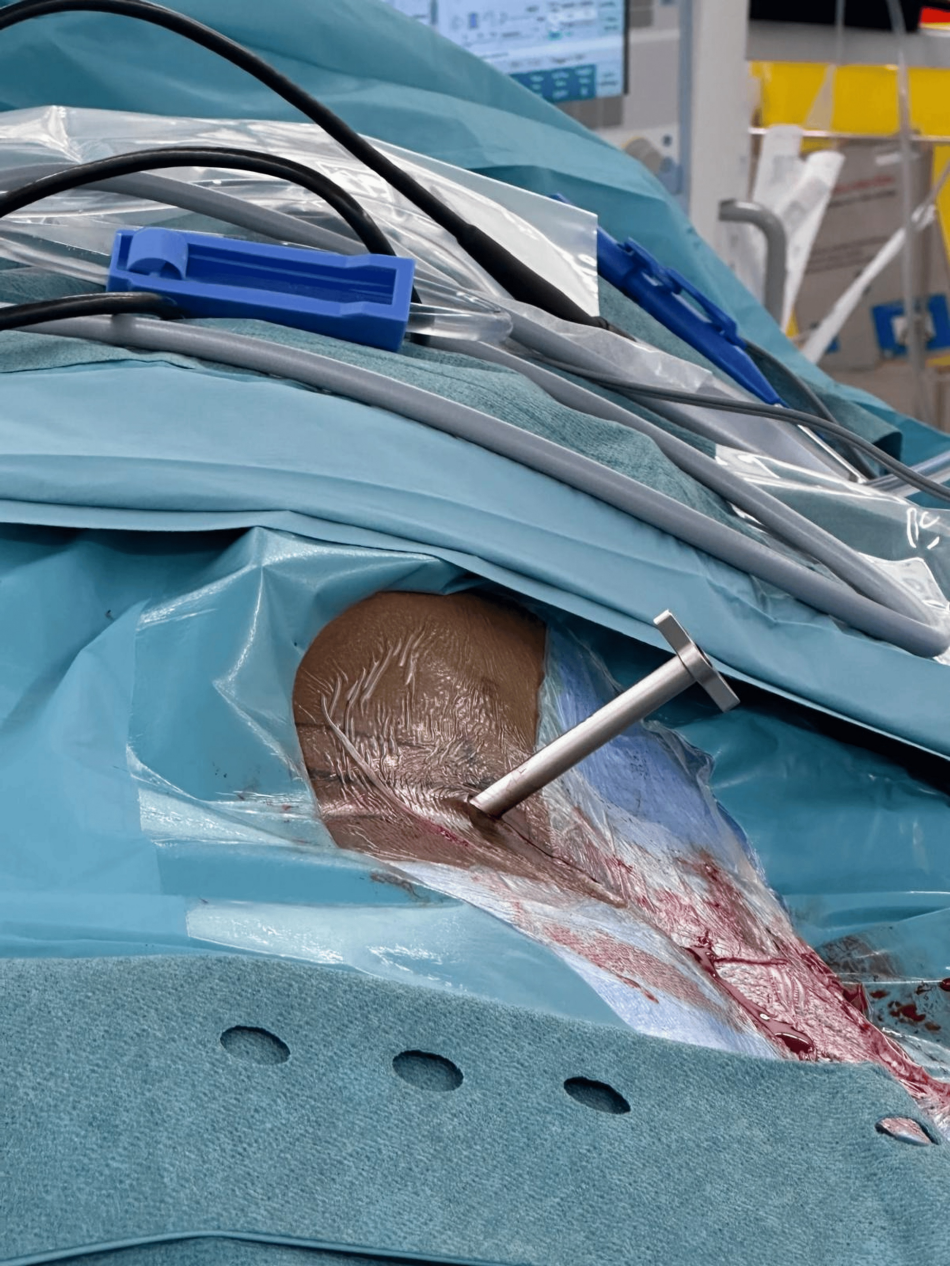
Serial dilators

Patients were positioned prone on a radiolucent operating table. The procedure was performed under general anesthesia combined with regional anesthesia for postoperative pain management. The operating surgeon stood on the herniated disc side with the scrub nurse and instruments table. The endoscope tower with monitor and the C-arm fluoroscope were placed on the opposite side. The entry point was made around 10 to 12cm lateral to the midline (Figure 1). An 18-gauge needle was inserted using fluoroscopy following local anesthetic infiltration. The tip of the needle was placed between medial and lateral pedicular lines on the anteroposterior (AP) and at the posterior vertebral line on lateral views (Figure 2). The needle was advanced using X-rays to reach the target point which on the lateral view was the upper corner of the caudal vertebra and the medial pedicle wall on the AP view (Figure 3). Then serial dilatation procedures were done using dilators of different sizes (Figure 4).

**Figure 5:**
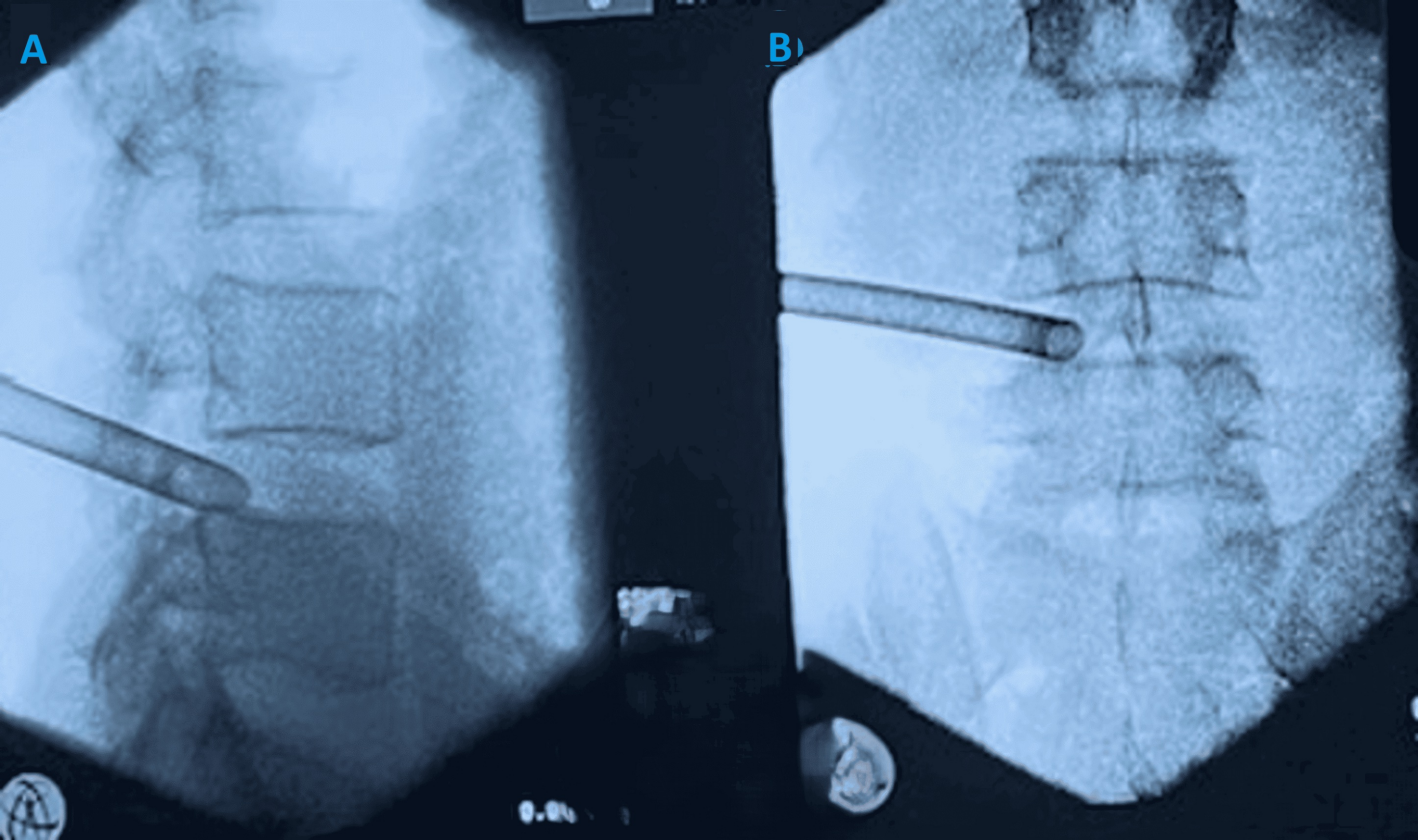
X-rays to confirm the position of the dilator in lateral (A) and AP (B) views. AP: Anteroposterior

A check X-ray was done to confirm the position of the working channel before introduction of the endoscope and camera (Figure 5).

**Figure 6:**
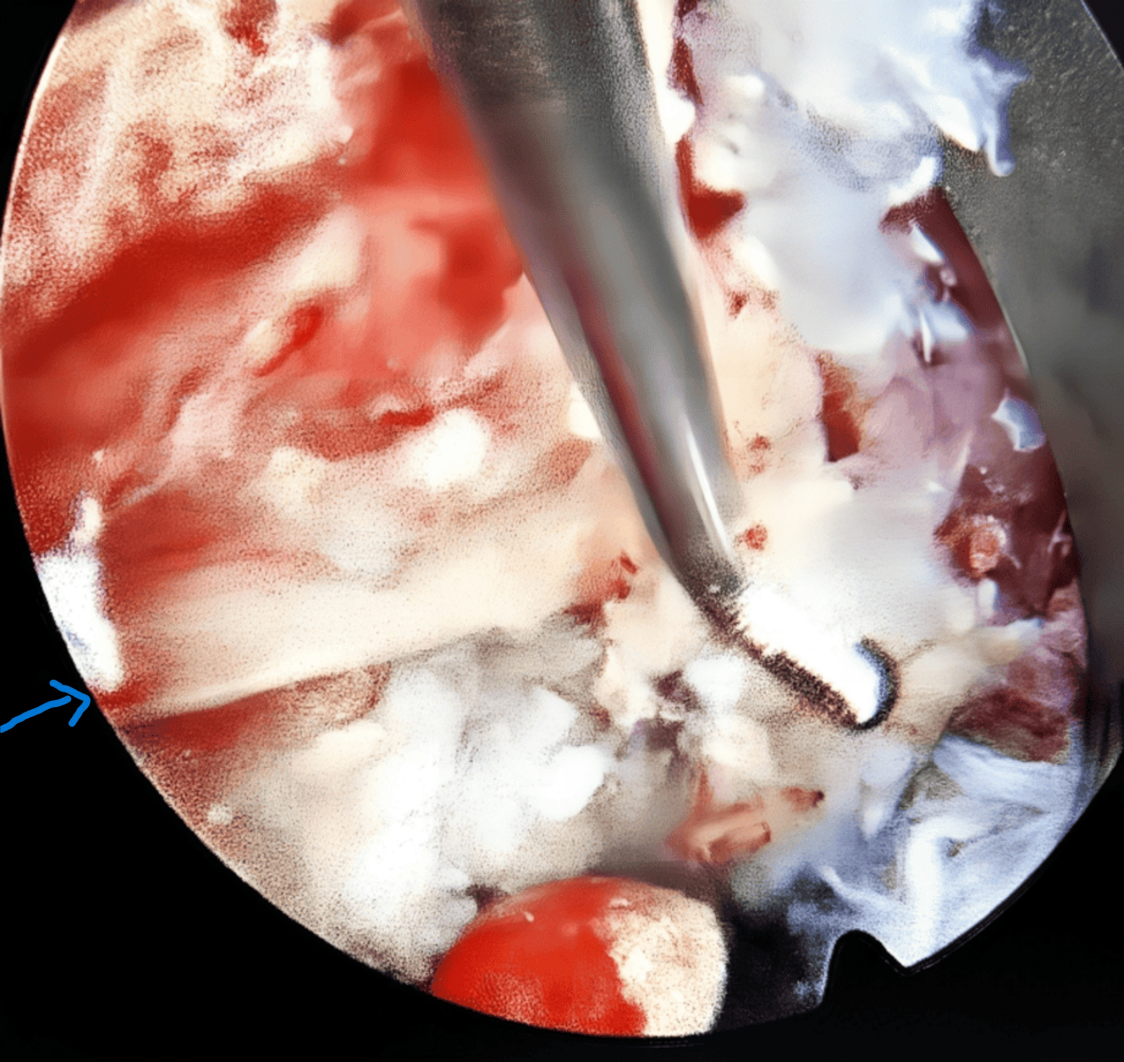
Nerve root decompression (blue arrow)

**Figure 7:**
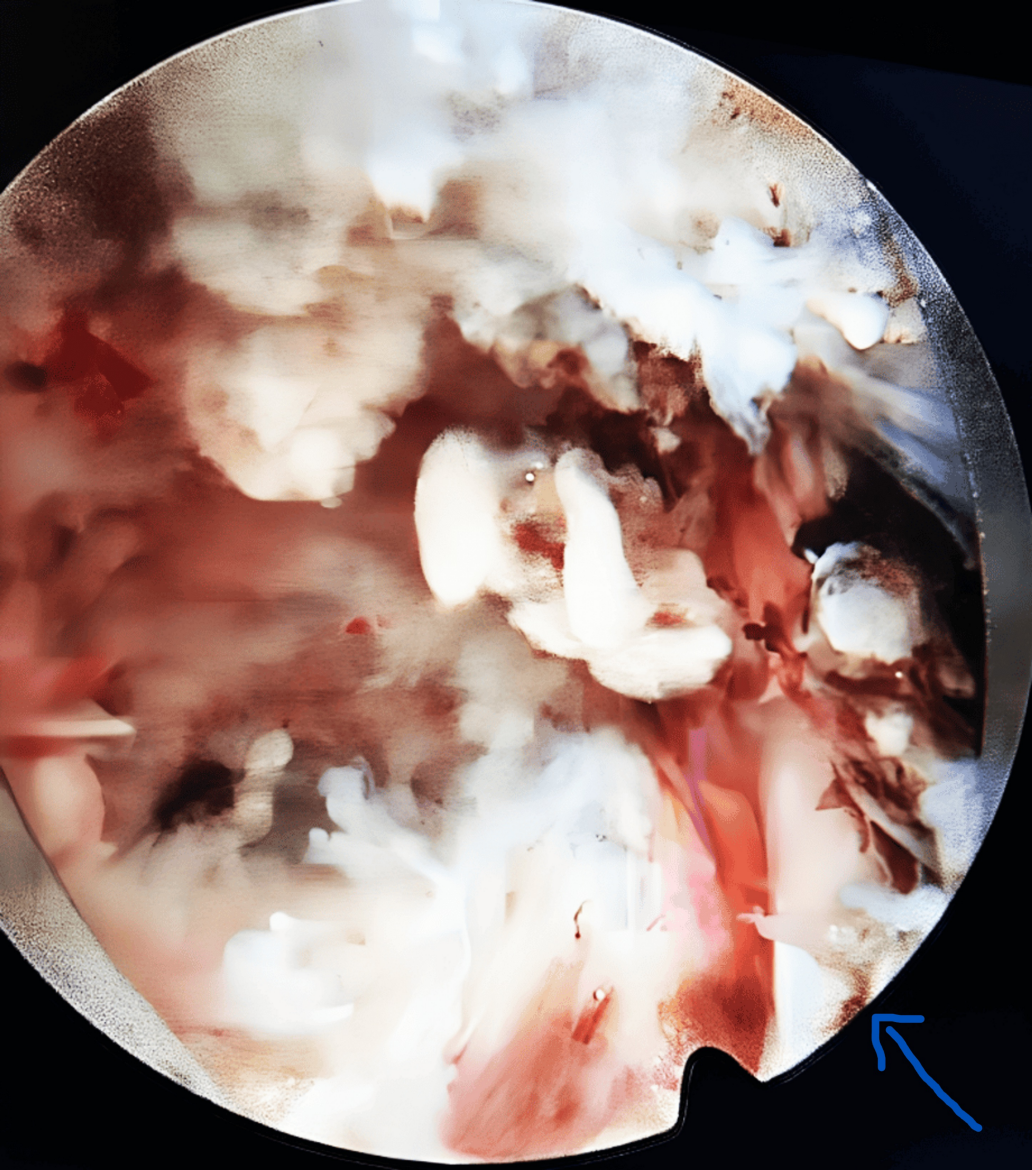
Nerve root release (blue arrow)

The herniated disc material was removed with the grasper. After removing all herniated disc fragments, an endoscopic view was done to confirm the release of pressure and free mobility of the affected nerve root (Figures 6, 7). After removing the instruments, the skin was closed with a single skin suture.

## Results

**Figure 8:**
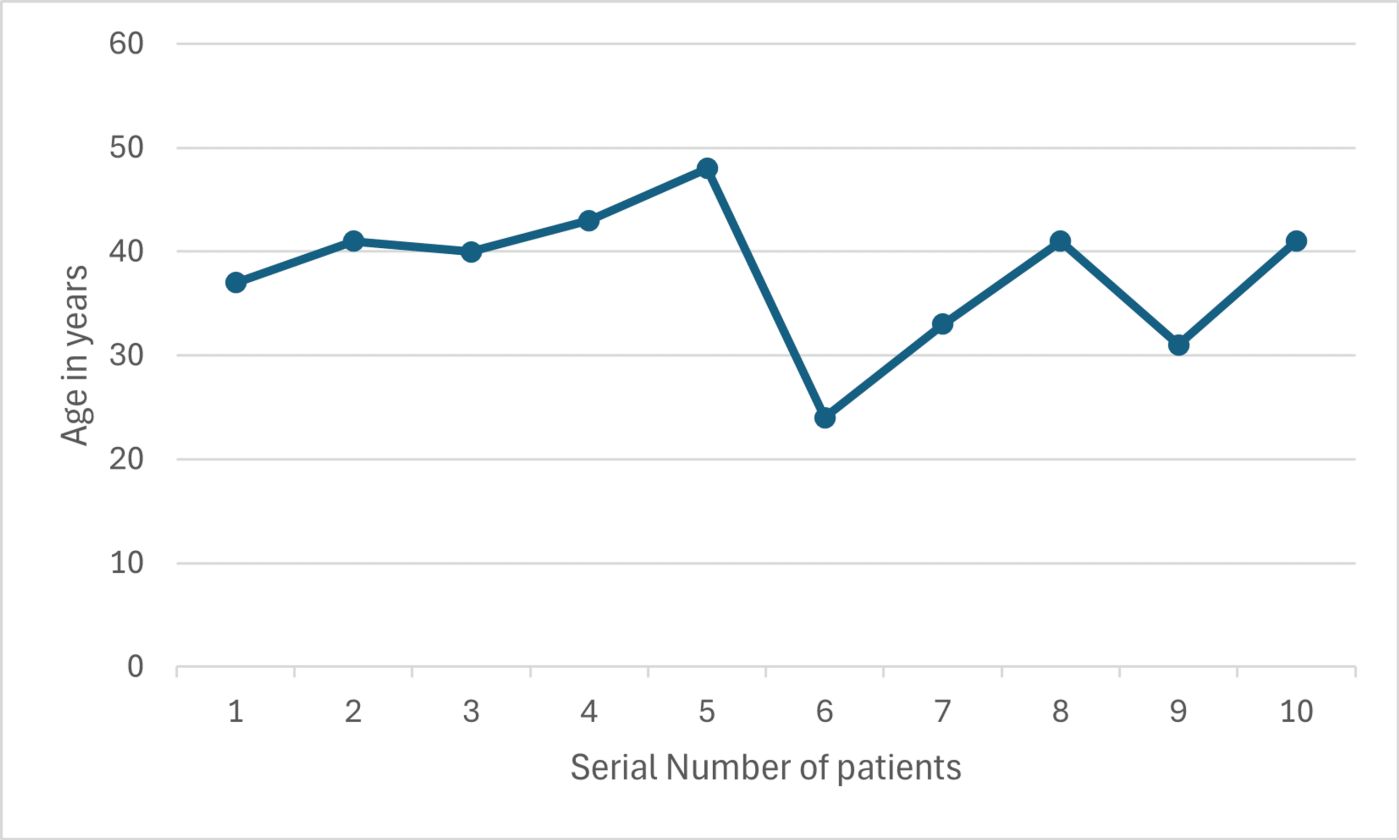
Patients' ages

Ten cases underwent TELD at the (L3/L4 - 1 case, L4/L5 - 8 cases and L5/ S1- 1 case) levels. All patients were male, and the mean age was 37.9 years, ranging from 24 to 48 years (Figure 8). No intraoperative complications were encountered like dural tear and nerve root injury. In addition, no infection was noted in these patients.

**Figure 9:**
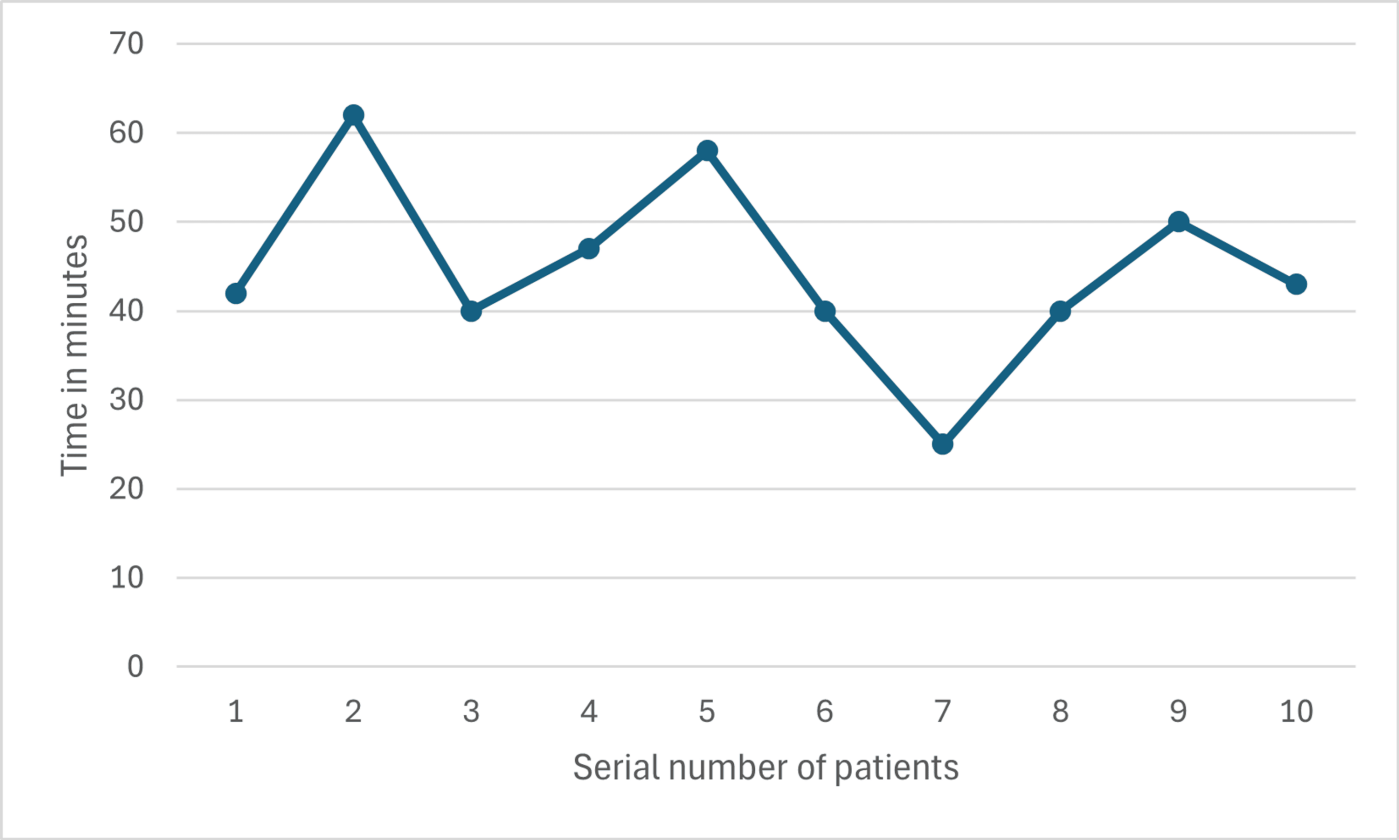
Preparation time

**Figure 10:**
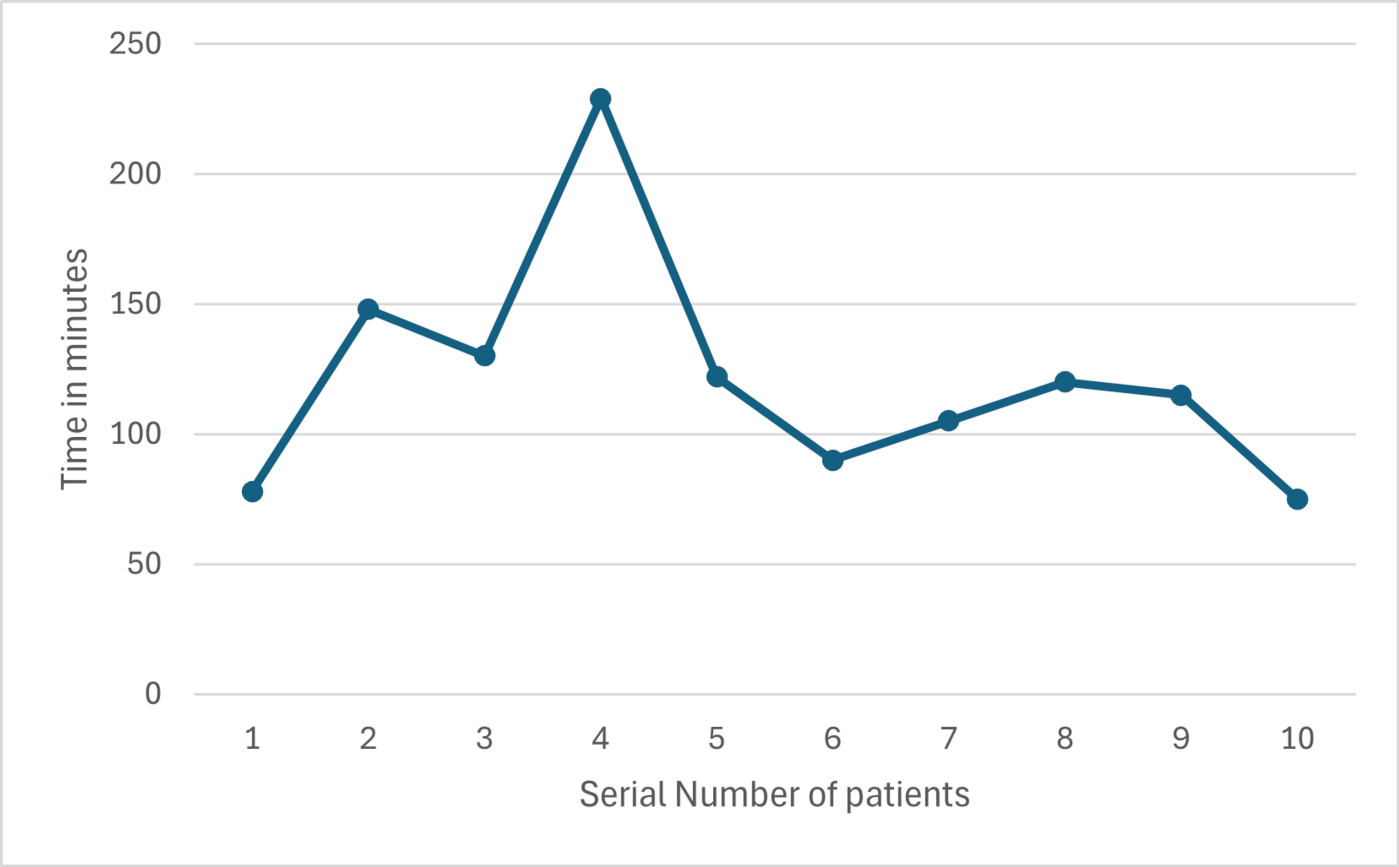
Surgery time

**Figure 11:**
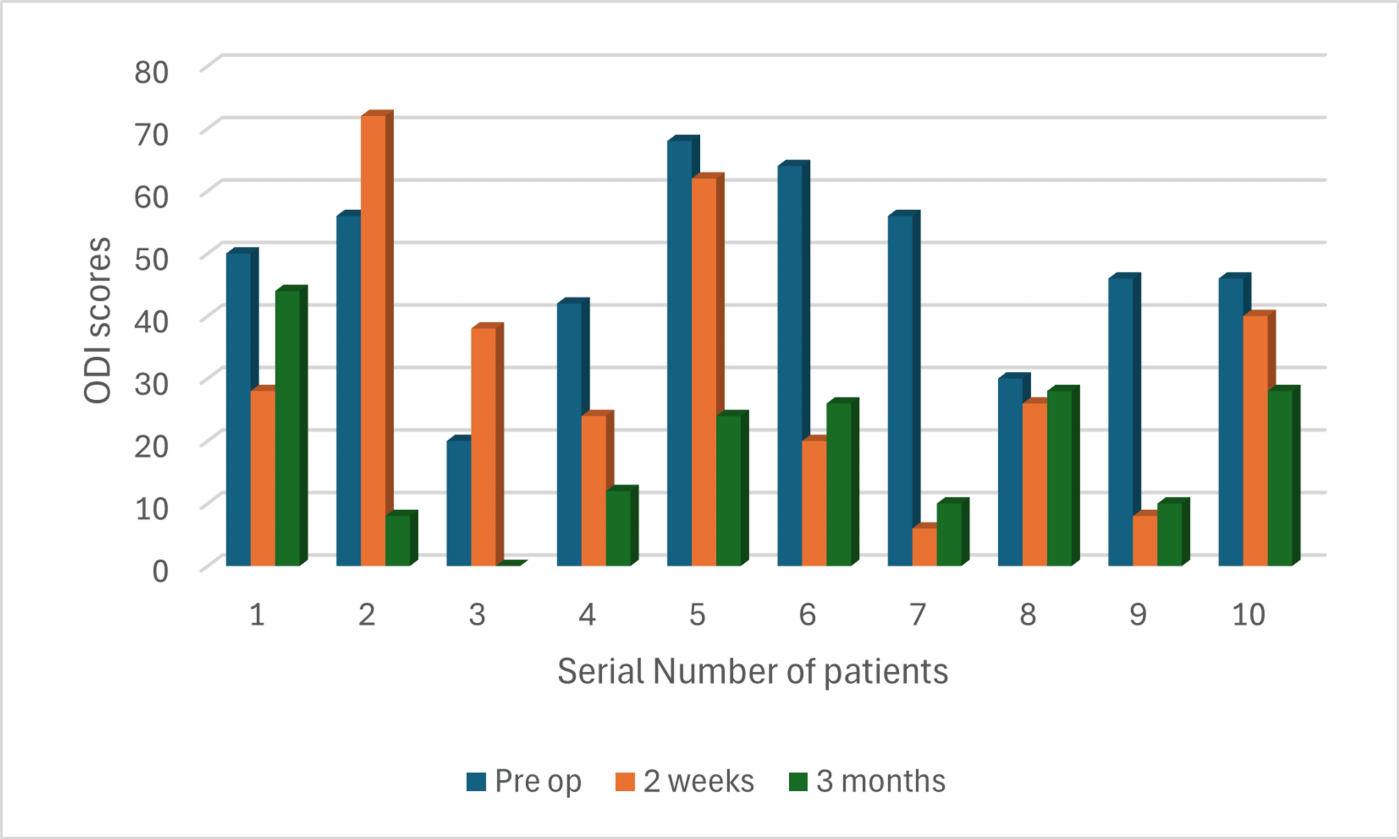
ODI scores ODI: Oswestry Disability Index

**Figure 12:**
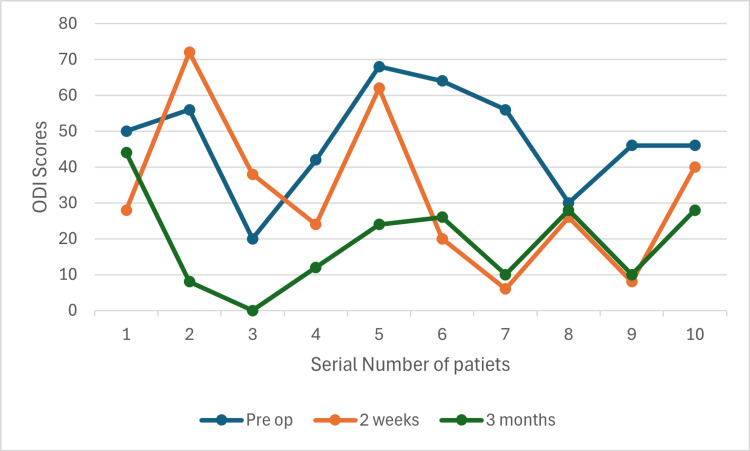
ODI score comparison ODI: Oswestry Disability Index

Preparation time varied between maximum of 62 minutes and minimum of 25 minutes (Figure 9), while the surgery time was between 75 and 225 minutes (Figure 10). The first and eighth operated cases did not show any significant improvement in the ODI scores (Figures 11, 12). Conversely, the remaining operated cases had a significant decrease in their ODI scores especially, at three months postoperatively (Figures 11, 12).

## Discussion

Preparation time and surgery time were the two main tools used to assess the learning curve of the operating surgeons in this study. We noticed that the preparation time remained almost stable with no significant changes, which indicates that no special challenges were encountered in preparing these patients for surgery. On the other hand, a downward trend was noticed in surgery time after the fourth case which may indicate the initial learning curve. Many previous studies tried to materialize the number of cases to attain a plateau of the learning curve [2,9,14,15]. However, others deny the existence of such numbers [16]. We found a relatively steeper learning curve in our study.

The ODI scores were used to determine the patients' satisfaction and the overall outcome of the surgeries. It was observed that there was an obvious decline in these scores in the majority of our cases, which is in accordance with many previously published studies [17,18]. However, these scores were mostly noticed to be high at two weeks and declined at three months postoperatively. These findings may indicate a relatively late relief of the initial symptoms.

Two of our cases were started initially as TELD, but they were converted intraoperatively to Md, so in our view, all those patients planned for TELD should also be consented and counselled for the possible conversion to Md, particularly at the beginning of the surgeon's career.

All our operated cases were discharged the next morning after they were adequately ambulated postoperatively. Although our case series included only the initial 10 cases, which is a very limited number to draw these conclusions, these findings indicate the initial trends that could be re-tested with larger studies with an increased number of patients.

## Conclusions

With proper selection of the patients, TELD is an effective procedure with favorable outcomes in terms of patient's satisfaction and overall improvement. However, the satisfactory results may be apparent relatively late. There is a learning curve of the surgeon for the procedure, which may cause the initial prolonged surgical times in these cases. However, the learning curve is not usually correlated with the patient's satisfaction and outcomes.
